# Comparison of Productivity and Quality of Three Perennial Ryegrass Cultivars and Their Mixture in Response to Nitrogen Fertilization and Grass-Legume Mixtures

**DOI:** 10.3390/plants13223130

**Published:** 2024-11-07

**Authors:** Gintarė Šidlauskaitė, Monika Toleikienė, Žydrė Kadžiulienė

**Affiliations:** Institute of Agriculture, Lithuanian Research Centre for Agriculture and Forestry, Instituto al. 1, Kėdainiai District, LT-58344 Akademija, Lithuania; monika.toleikiene@lammc.lt (M.T.); zydre.kadziuliene@lammc.lt (Ž.K.)

**Keywords:** forage composition, monoculture, multi-species, *Lolium perenne* L., sward persistence

## Abstract

We conducted a four-year cutting experiment on herbage yield, with three years focused on testing the effect of perennial ryegrass (PR) cultivars ‘Elena DS’, ‘Raminta’, and ‘Verseka’, along with a cultivar mixture and compositions with white clover (WC) and red clover (RC) diversity, on crude protein (CP), modified acid detergent fibre (MADF), neutral detergent fibre (NDF), water-soluble carbohydrates (WSC), and dry matter digestibility (DMD) content. PR cultivars and cultivar mixtures were sown alone (N_150_), and the PR ‘Elena DS’ and PR cultivar mixtures were also sown with each WC, or WC and RC (N_0_). The average four-year herbage productivity was the lowest in PR ‘Elena DS’ the and cultivar mixture/WC, followed by all of the pure PR swards, and the highest in PR ‘Elena DS’ and the cultivar mixture/WC+RC; however, the PR ‘Elena DS’/WC+RC mixture had the highest legume proportion and CP content. There was less NDF but also WSC in swards with legumes, but higher CP content than in pure PR swards; however, the highest CP content was in mixtures with RC. Among the grasses, PR ‘Verseka’ had lower NDF contents and a higher WSC than PR ‘Elena DS’, ‘Raminta’, and the cultivar mixture. Overall, this study revealed significant differences in the productivity of PR cultivars with a potential yield difference of up to 1.7 t ha^−1^.

## 1. Introduction

The primary challenges facing the agricultural sector are to reduce production costs, meet product safety and quality requirements, and increase production profitability. An intensive agricultural system directs the farmer more towards crop fertility and less towards soil productivity and environmental conservation [1]. Meanwhile, increased anthropogenic activity has a significant negative impact on the stability of the entire ecosystem [2] and the long-term productivity of the soil. One of the undesirable phenomena caused by the application of intensive agricultural technologies is the decrease in soil organic matter, as less organic matter enters the soil than decomposes. It is known that an increase in soil organic matter content can be influenced by the cultivation of perennial plants [3,4], especially leguminous grasses [5], balanced fertilization with organic and mineral fertilizers, intercropping [3], and conservation tillage.

It is already known that short-term grasslands can be used not only as fodder for livestock, but also incorporated into crop rotations to improve soil structure [6], increase moisture retention, enhance biological activity [7], and reduce erosion risk. Additionally, grasslands contribute to increasing the accumulation of organic matter [8], help regulate nitrogen, phosphorus, and other nutrient cycles to improve soil fertility [9], and reduce the risk of leaching into groundwater systems. Moreover, the direct benefit of grasslands in preserving biodiversity, enriching soil organism communities, and indirectly improving subsequent crop yields has also been acknowledged.

Incorporating legumes into swards systems positively impacts herbage production and quality [10]. Red and white clovers are particularly important in European grasslands. The biological nitrogen fixation capacity of legumes reduces the need for mineral nitrogen fertilizer. Numerous studies have shown that herbage production can be maintained or increased with the presence of white clover in the sward, even with reduced mineral nitrogen fertilizer input [11,12]. Red clover is notable for producing large quantities of high-quality silage with minimal to no mineral nitrogen fertilizer [13], making it a crucial component of grassland-based production systems [14]. Legume incorporation in grasslands has been shown to maintain or enhance herbage quality [15] at lower nitrogen fertilizer application rates compared to grass-only swards, thereby boosting animal production. For instance, the digestibility of grass-clover swards could be higher [16] than that of grass-only swards at low chemical nitrogen fertilizer input.

The majority of plant biomass from swards is utilized in animal husbandry, either through direct grazing or by making hay for winter feed. This study aims to evaluate how individual perennial ryegrass (*Lolium perenne* L.) cultivars and their mixture contribute to the overall productivity and quality parameters of swards, considering the ecosystem services provided by swards, with the goal of reducing the use of mineral nitrogen (N) fertilizers. Mixtures of perennial ryegrass cultivars can enhance forage production by optimizing specific functional traits within the combinations [17]. In our study, we selected three Lithuanian tetraploid perennial ryegrass cultivars, which often exhibit higher biomass production. Due to their thicker and more robust leaves, tetraploid cultivars may be more drought resistant [18]. Additionally, the increased chromosome number can result in higher sugar content in the leaves, making the forage more nutritious. However, there is a need to explore the potential of cultivar mixtures [19] to maximize the benefits of perennial ryegrass in various ecological conditions.

The aim of this study was to determine the effects of forage diversity and the nutritive quality of swards in a four-year monoculture and multi-species swards experiment with and without nitrogen application, using a cutting regime. Swards were cut over a four-year period, while qualitative analyses were performed across three years of the experiment. In addition to annual herbage productivity, the study also presents the detailed botanical composition of the swards, which illustrates the influence of legume grasses on overall sward productivity and quality. The results revealed significant differences in yield between perennial ryegrass cultivars across different years of sward use, with variations ranging from 298 to 1700 kg ha^−1^. These findings provide valuable insights into optimizing grassland management and highlight the positive impact of legumes on forage quality, yield, and weed suppression. The study’s insights contribute to more efficient forage production systems for sustainable agricultural practices.

## 2. Results

### 2.1. Annual Yield

**Figure 1:**
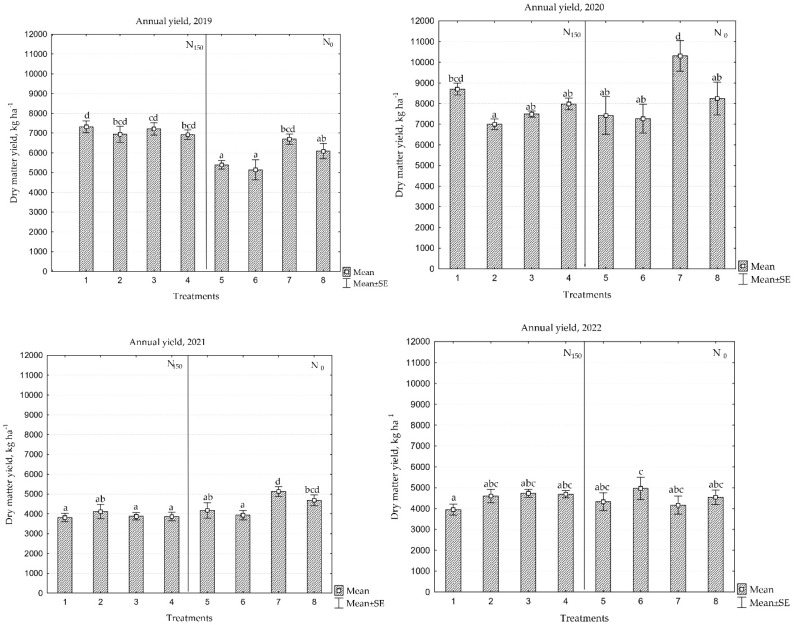
Annual dry matter yield during the 2019–2022 period. Treatments: 1, perennial ryegrass (PR) cv. ‘Elena DS’; 2, PR cv. ‘Raminta’; 3, PR cv. ‘Verseka’; 4, PR cultivar mixture; 5, white clover + PR cv. ‘Elena DS’; 6, white clover + PR cultivar mixture; 7, white and red clover + PR cv. ‘Elena DS’; and 8, white and red clover + PR cultivar mixture. Level of N fertilizer per year: 1–4 treatments (N_150_), 5–8 (N_0_); different letters indicate significant differences between the treatments (*p* < 0.05).

A statistically significant difference in dry matter yield (DMY) was observed when comparing swards with different species compositions (*p* < 0.05), except for 2022, where no significant differences were found between the treatments (Figure 1). In the first year of sward use, all four monoculture swards, which included different PR cultivars and a cultivars mixture (N_150_), were significantly more productive than the swards with diverse species composition (N_0_); however, it was observed that white and red clovers together in the mixture with PR cv. ‘Elena DS’ could substitute for mineral N fertilization even in the first year, as no statistically significant differences in productivity were found between these treatments. In the first year of sward use, drought conditions were recorded, leading to lower overall sward productivity compared to the second year, which was favourable for sward growth and development.

The productivity of the PR cultivar ‘Elena DS’ was 1378 kg ha^−1^ higher under favourable conditions, while the PR cultivar ‘Raminta’ showed no difference. The PR cultivar ‘Verseka’ had a productivity increase of 280 kg ha^−1^, and the PR cultivar mixture increased by 1064 kg ha^−1^. In dry years, a potential difference of up to 380 kg ha^−1^ may occur between the perennial PR ‘Elena DS’, ‘Raminta’, and ‘Verseka’, whereas in favourable growing years, the difference can be up to 1700 kg ha^−1^. Additionally, the productivity of the PR cultivar mixture in both the first and second years did not exceed that of the most productive PR cultivar, the ‘Elena DS’ sward.

**Figure 2:**
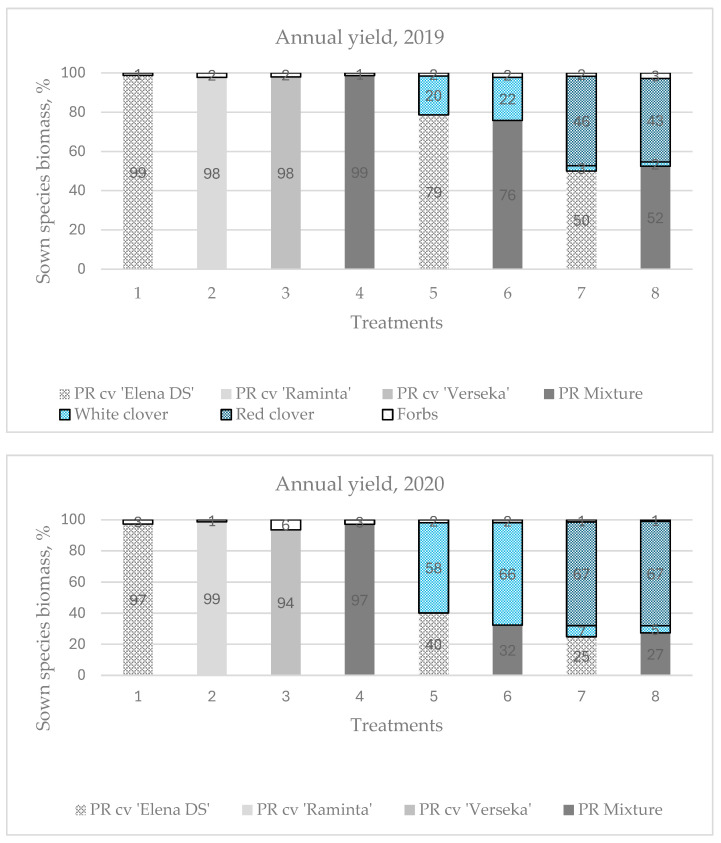

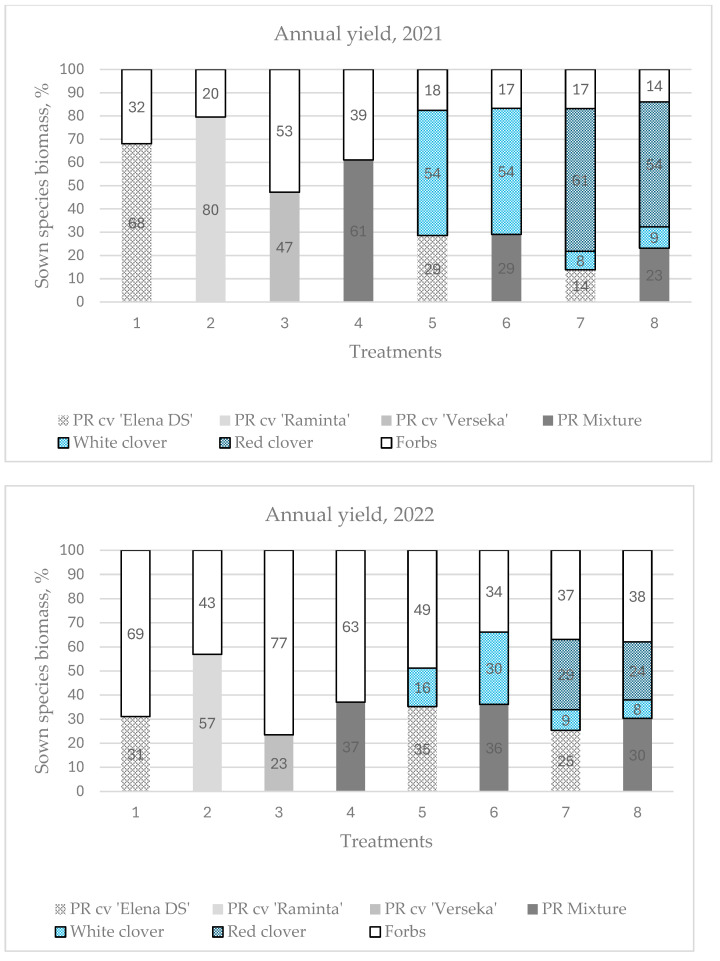
Biomass contribution (%) of the main species in each sward in the four-year experimental period (2019–2022). PR, perennial ryegrass. Level of N fertilizer per year: 1–4 treatments (N_150_), 5–8 (N_0_).

The two-year average yield of the PR cultivar ‘Elena DS’ reached 8010 kg ha^−1^, while the productivity of PR ‘Raminta’ was the lowest among the cultivars at 6970 kg ha^−1^. The yield of PR ‘Verseka’ was 7353 kg ha^−1^, and the PR cultivar mixture yielded 7449 kg ha^−1^. In the third and fourth years of sward use, the productivity of monoculture swards (N_150_) decreased by an average of 43%, primarily due to the age of the swards. A significant increase in the proportion of forbs was observed in these swards, contributing between 20% and 53% of the total sward productivity in 2021 and rising to between 43% and 77% in 2022, respectively (Figure 2).

This study also revealed that the productivity of PR ‘Elena DS’ and the PR cultivar mixture differed significantly when using N_150_ and legume grasses. A comparison between PR ‘Elena DS’ and the PR cultivar mixture, focusing on their ability to grow with leguminous grasses and reduce or eliminate the use of mineral N fertilizers in multi-species swards, showed significant results. The PR ‘Elena DS’ mixture with white and red clovers had an average yield of 8501 kg ha^−1^ in the first two years, which was 5.8% higher than the yield of the fertilized PR ‘Elena DS’ monoculture sward. In the third and fourth years, the productivity of this sward also decreased by 45%; however, compared to monoculture swards, the proportion of forbs was nearly 50% lower. Meanwhile, the productivity of the PR cultivar mixture with white and red clovers did not exceed that of the most productive fertilized monoculture sward. In swards containing only white clover, differences in productivity were also observed between PR ‘Elena DS’ and the PR cultivar mixture. In all years, the yield of the PR ‘Elena DS’ was higher than the PR cultivar mixture, except for in 2022; however, the overall sward productivity in these cases was lower compared to the other swards.

### 2.2. Botanical Composition and Species Diversity

The botanical composition of swards is a crucial factor determining their productivity and forage quality. According to the data from our experiment, the botanical composition of the swards varied across different years of sward use (Figure 2). In most cases, legume grasses were predominant in multi-species swards containing PR ‘Elena DS’ and the PR cultivar mixture. No significant differences in the botanical composition changes of perennial grasses were observed when evaluating individual years in these swards. However, it was observed that a greater diversity of legume species within the sward reduced the proportion of perennial grasses, influencing the annual productivity of the swards. In the first year of sward use, PR ‘Elena DS’ accounted for 79% of the total sward biomass, while the PR cultivar mixture made up 76% when grown with white clovers. In mixtures with both white and red clovers, these percentages decreased to 50% and 52%, respectively. By the second year, the contribution of the PR ‘Elena DS’ further decreased from 40% and 32% to 25% and 27%, respectively. In the third year, it dropped from 29% to 14% and 23%, and in the fourth year, it declined from 35% and 36% to 25% and 30%, respectively. As the botanical composition of the swards changed, the increasing proportion of legume species generally led to higher annual sward productivity, greater crude protein (CP) content, and nearly 50% lower forb content compared to monoculture swards. Therefore, monoculture swards that are additionally fertilized with N_150_ can remain productive for up to two years. In subsequent years, the quantity of forbs significantly increases, with notable differences observed among cultivars.

The average forbs content over the third and fourth years of swards use for PR ‘Elena DS’ was 48%, which was higher than the 30% for PR ‘Raminta’, but lower than the 56% for ‘Verseka’. The cultivar mixture had a similar forbs content to ‘Elena DS,’ at 45%. PR ‘Elena DS’ was the most productive in the first two years of sward use (8010 kg ha^−1^), whereas ‘Raminta’ was more productive in the third and fourth years (4360 kg ha^−1^). The average difference in dry matter yield between these cultivars over four years was only 282 kg ha^−1^, highlighting not only the cultivars’ potential to accumulate high biomass, but also their ability to maintain sward quality with lower levels of forbs throughout the growing seasons. Therefore, it is essential to investigate not only the performance of individual perennial grasses, but also the growth potential of their cultivars under changing climatic conditions.

### 2.3. Variation in Forage Yield Across Experimental Cuts and Years

**Figure 3:**
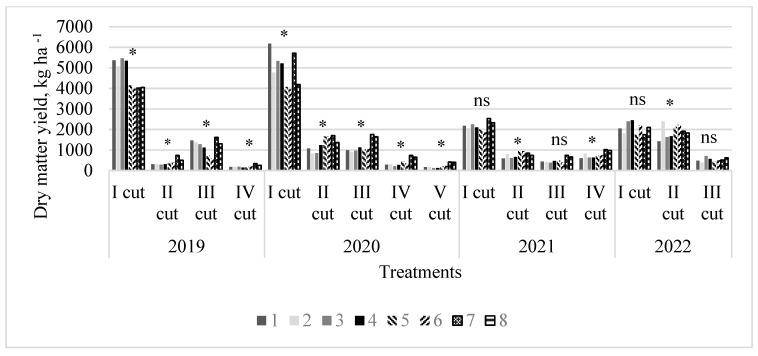
Dry matter yield of swards during the experimental years in different cuts in the four-year experimental period (2019–2022). Treatments: 1, perennial ryegrass (PR) cv. ‘Elena DS’; 2, PR cv. ‘Raminta’; 3, PR cv. ‘Verseka’; 4, PR cultivar mixture; 5, white clover + PR cv. ‘Elena DS’; 6, white clover + PR cultivar mixture; 7, white and red clover + PR cv. ‘Elena DS’; 8, white and red clover + PR cultivar mixture. Level of N fertilizer per year: 1–4 treatments (N_150_), 5–8 (N_0_); * values with asterisks indicate significant differences (* *p* < 0.05), and ns indicates non-significant difference between the treatments in different cuts.

The number of cuts performed in individual years of sward use depended on the meteorological conditions. The second year of sward use was particularly favourable for sward growth and development, resulting in five cuts (Figure 3). In subsequent years, the number of cuts decreased due to impaired grass growth. In the first two years of swards use, statistically significant differences (*p* < 0.05) were found between the treatments in all cuts. In contrast, in the third and fourth years, differences in sward productivity were observed in many cases, but they were not statistically significant. These results were likely influenced by a significant increase in forbs content in monoculture swards; in 2021, forbs content rose from 15% in the first cut to 65% in the final cut, and in 2022, from 67% to 78%. In the first year of sward use, monoculture swards were the most productive in the first cut, with the PR cultivar ‘Verseka’ showing the highest yield. However, no significant differences were found between this cultivar, other cultivars, or the cultivar mixture. Grass-legume swards had significantly lower productivity in the first cut compared to monoculture swards, but these differences varied when evaluating subsequent cuts and overall annual yield. A pattern was observed where the multi-species sward with PR ‘Elena DS’ combined with white and red clovers was not only the most productive in terms of the average annual dry matter yield over four years—reaching 6695 kg ha^−1^ in 2019, 10,308 kg ha^−1^ in 2020, 5133 kg ha^−1^ in 2021, and 4167 kg ha^−1^ in 2022—but also consistently demonstrated the highest productivity in all cuts from the second cut of 2019 to the fifth cut of 2020, and in 2021, with the exception of the second cut. The stable productivity of this grass-legume sward across all cuts and over the entire evaluation period indicates its potential for effective long-term use, ensuring consistent yields even under changing environmental conditions. The comparison indicates that, while monoculture swards remain productive only for the first two years, with a notable decline in productivity due to forbs in the third and fourth years, the grass-legume sward’s consistent performance throughout the evaluation period suggests it offers a more sustainable solution. The grass-legume sward’s ability to maintain stable yields over time, despite changing environmental conditions, highlights its advantage for long-term use compared to monocultures, which suffer from reduced productivity as forbs become more dominant.

However, the results indicate that the grass-legume sward consisting of the perennial ryegrass cultivar mixture with white and red clovers did not demonstrate the same level of consistent yield stability as the PR cultivar ‘Elena DS’. Despite incorporating leguminous species that are known for their beneficial effects on soil fertility and sward productivity, the single cultivar ‘Elena DS’ achieved superior performance in terms of yield stability. Furthermore, the mixture could have faced challenges related to the differential growth rates and nutrient requirements of the included cultivars, leading to less optimal performance.

### 2.4. Crude Protein

When evaluating crude protein (CP) content, significant differences were observed between different swards in individual cuts and across different years (Table 1). Comparing individual years based on the average CP content in the swards, it was observed that CP in the first cut tended to increase from 9.5% to 14.9% in the first and third years of sward use, respectively. These results indicate that in swards with diverse species composition, legume grasses require time to establish dominance, as the proportion of legumes in the total biomass also increased over the years. Furthermore, when comparing the three-year average CP content, the third cut accumulated 31.5% more CP than the first cut. Over the three-year period on average, in the first cut, the lowest CP content was in the monoculture of PR ‘Elena DS’ sward at 10.2%, while the highest was in the PR ‘Elena DS’/WC+RC mixture at 13.6%. In the third cut, these values corresponded to 14.9% and 19.5%, respectively.

**Figure 4:**
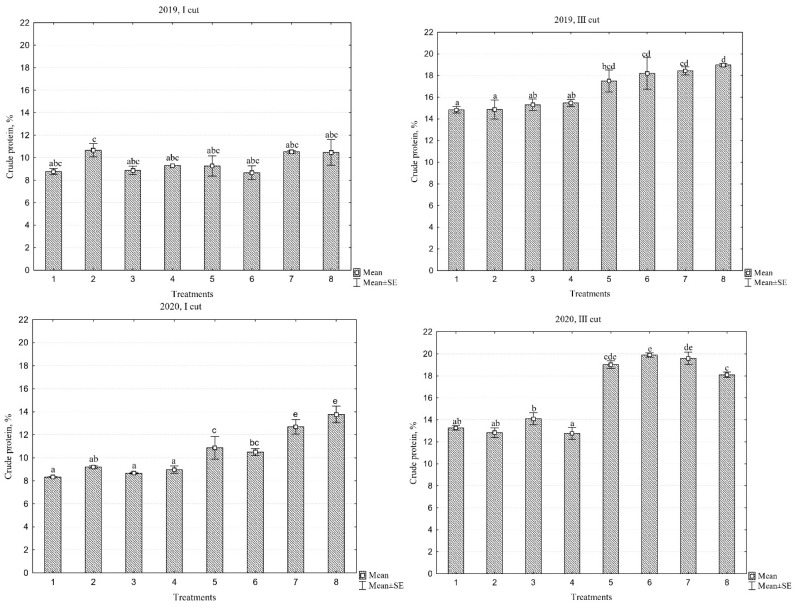

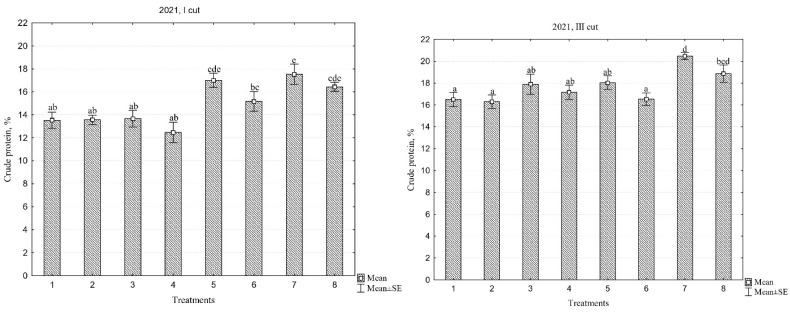
Crude protein content across different cuts during the three-year period (2019–2021). Treatments: 1, perennial ryegrass (PR) cv. ‘Elena DS’; 2, PR cv. ‘Raminta’; 3, PR cv. ‘Verseka’; 4, PR cultivars mixture; 5, white clover + PR cv. ‘Elena DS’; 6, white clover + PR cultivar mixture; 7, white and red clover + PR cv. ‘Elena DS’; 8, white and red clover + PR cultivar mixture. Level of N fertilizer per year: 1–4 treatments (N_150_), 5–8 (N_0_); different letters indicate significant differences between the treatments (*p* < 0.05).

The crude protein (CP) contents of the first and third cuts in 2019–2021 are shown in Figure 4. The data indicate that the average CP content, when evaluating the first cut, increased with each successive year of sward use; as the swards aged, the CP content also increased. When evaluating fertilized swards with different cultivars of PR and the PR cultivar mixture, no significant differences in CP content were observed in these swards across individual years of use. These findings indicate that the increase in CP content in different years of sward use was significantly influenced by the presence of legume grasses. In the first cut of each year, monoculture swards accumulated an average CP content ranging from 9.4% to 13.3%, while multi-species swards accumulated from 9.7% to 16.5%, respectively, in 2019 and 2021. In the third cut, the CP content was higher than in the first cut, when evaluating swards with different species compositions; in 2019, CP content ranged from 14.8 to 19%, in 2020 from 12.8 to 19.9%, and in 2021 from 16.3 to 20.5%, in the monoculture and multi-species swards, respectively.

### 2.5. Modified Acid Detergent Fiber

The data presented in Table 2 indicate that the different species compositions of the swards, different years, and their interaction had a significant effect on modified acid detergent fibre (MADF) content in the first and third cuts (Table 2). Evaluating different years, the highest MADF content was observed in the first year of sward use, with an average of 29.2% in the first cut. In contrast, a 24% reduction in MADF content was noted in the second and third years of sward use. Furthermore, in the third cut, unlike in the first cut, the MADF accumulated in 2019 was the lowest at 24.1%, while in the following years, there was an approximate 10% increase. Notably, in both the first and third cuts, the average MADF content over the three years was lowest in the PR ‘Elena DS’/WC+RC mixture, while the highest was found in the monoculture of PR ‘Elena DS’ sward.

**Figure 5:**
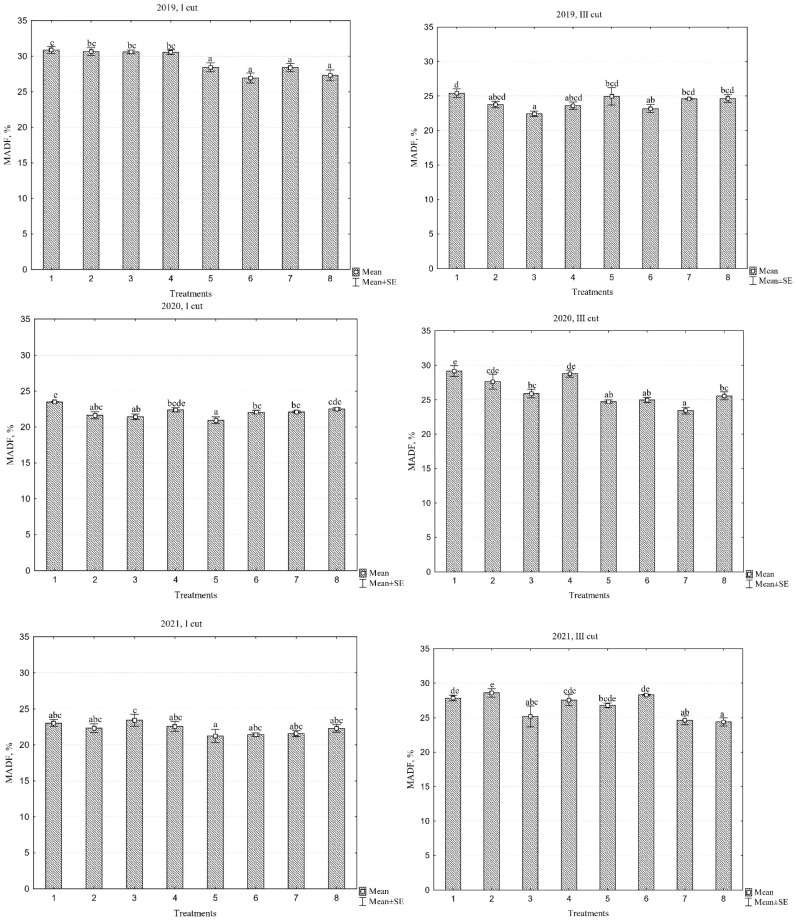
Modified acid detergent fibre content across different cuts during the three-year period (2019—2021). Treatments: 1. perennial ryegrass (PR) cv. ‘Elena DS’; 2, PR cv. ‘Raminta’; 3, PR cv. ‘Verseka’; 4, PR cultivar mixture; 5, white clover + PR cv. ‘Elena DS’; 6, white clover + PR cultivar mixture; 7, white and red clover + PR cv. ‘Elena DS’; 8, white and red clover + PR cultivar mixture. Level of N fertilizer per year: 1–4 treatments (N_150_), 5–8 (N_0_); different letters indicate significant differences between the treatments (*p* < 0.05).

Modified acid detergent fibre (MADF) indicates the indigestible portion of plant material, such as cellulose and lignin, in the sward. Lower MADF values generally suggest higher digestibility and, therefore, better forage quality. When evaluating sward quality, a lower MADF content is desirable, as it correlates with better nutrient availability and energy content for livestock. For high-quality forage, the MADF values should typically range between 25 and 35%. Values above this range suggest lower digestibility, which can limit the feed intake and nutritional benefits for animals. When comparing different PR cultivars and the PR cultivar mixture, PR ‘Verseka’ tended to have the lowest MADF values, though not always significantly (Figure 5). These differences were most notable in the third cut, with MADF ranging from 22.4% to 25.9% across years, while PR ‘Elena DS’ had values between 25.4% and 29.2%.

The data also indicate that the overall average MADF content in swards was significantly higher during the first year of use, specifically in the first cut, compared to the following years. Nevertheless, the forage quality of all swards remained high, as the maximum recorded MADF value did not exceed 30.8%. Furthermore, the MADF values in multi-species swards were lower than in monoculture swards; however, in most cases, these differences were not statistically significant.

### 2.6. Neutral Acid Detergent Fiber

The statistical data presented in Table 3 indicate that no significant differences were found in the third cut across the different years. Additionally, no interaction between years and different treatments was observed in the first cut. However, significant differences between treatments were identified in all cuts throughout all years of sward use. In the first cut, the overall average NDF content in the swards tended to decrease from 56.3% in the first year of sward use to 38.2% in the third year; meanwhile, in the third cut, the differences were not significant between years, but the values ranged from 44.5% to 43.7%. When evaluating the quality of individual swards over the three-year period, the NDF content, similar to that of MADF, was lowest in the PR ‘Elena DS’/WC+RC mixture and highest in the monoculture of PR ‘Elena DS’ sward, at 41.8% and 50.4%, respectively.

**Figure 6:**
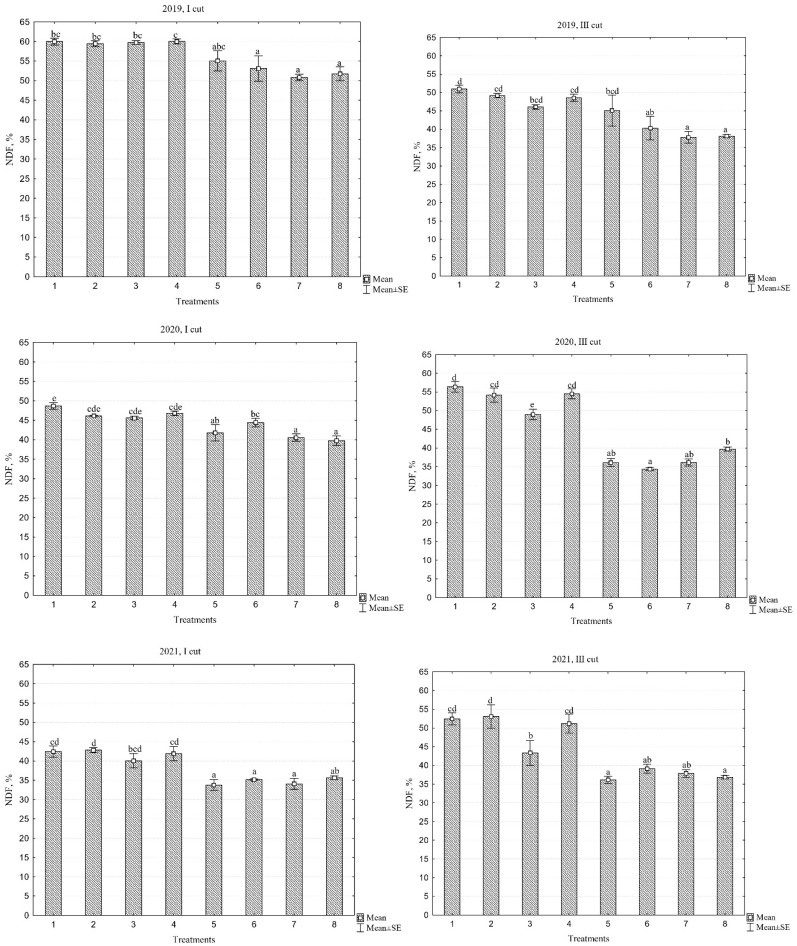
Neutral detergent fibre content across different cuts during the three-year period (2019–2021). Treatments: 1, perennial ryegrass (PR) cv. ‘Elena DS’; 2, PR cv. ‘Raminta’; 3, PR cv. ‘Verseka’; 4, PR cultivar mixture; 5, white clover + PR cv. ‘Elena DS’; 6, white clover + PR cultivar mixture; 7, white and red clover + PR cv. ‘Elena DS’; 8, white and red clover + PR cultivar mixture. Level of N fertilizer per year: 1–4 treatments (N_150_), 5–8 (N_0_); different letters indicate significant differences between the treatments (*p* < 0.05).

Although the MADF indicators for both monoculture and multi-species swards did not reach the critical threshold that would impair forage digestibility (Figure 5), monoculture swards were still found to be less digestible and less palatable for animal intake. Nitrogen fertilizers had a significant impact on the increase in NDF content in swards (Figure 6). Monoculture swards that were fertilized generally exhibited significantly higher NDF values than multi-species swards, indicating lower forage palatability. When comparing different years, it was found that in the first year of sward use, the NDF values in the first cut were significantly higher than in the second or third years. However, no significant differences between years were observed in the third cut.

The data also indicate that, in the first cut, the NDF values of multi-species swards were lower than those of monoculture swards by 11.7%, 11.1%, and 16.9% in 2019, 2020, and 2021, respectively, and by 17.2%, 31.6%, and 25% in the third cut. When evaluating different PR cultivars, it was noted that there were differences in NDF values among the cultivars, although in most cases, these differences were not significant. However, the PR cultivar ‘Verseka’ exhibited better palatability compared to other PR cultivars.

### 2.7. Water-Soluble Carbohydrates

The data presented in Table 4 indicate that the different species compositions of the swards, different years, and their interaction had a significant effect on water-soluble carbohydrates (WSC) content in the first and third cuts. Evaluating the WSC content in individual years, it was observed that the overall swards average WSC content in the first cuts was highest, reaching an average of 26.8% in the second year of sward use. In contrast, a nearly 50% reduction in WSC was noted in the third cut when considering different swards average data. When analyzing the three-year average data, the lowest WSC contents, averaging 16.1%, were found in the PR ‘Elena DS’/WC+RC mixture and the PR cultivar mixture/WC+RC. The highest WSC content was 23% in the monoculture sward with PR ‘Verseka’ during the first cut, while in the third cut, the corresponding values were 6.8% in the mentioned mixtures and 12% in the monoculture sward with PR ‘Raminta’. Overall, monoculture swards provided better energy availability for livestock compared to swards with diverse species compositions.

WSC in forage grasses are an important indicator of their nutritional value. WSC includes simple sugars such as glucose, fructose, and sucrose, as well as some polysaccharides. A high WSC content in forages is often associated with good quality, as it indicates that the plants are nutritious and provide more energy for livestock. Maintaining forage at a high-quality level often requires the WSC to be at least around 10% of dry matter (DM) yield. Typically, WSC in forages ranges between 10% and 20% of DM.

**Figure 7:**
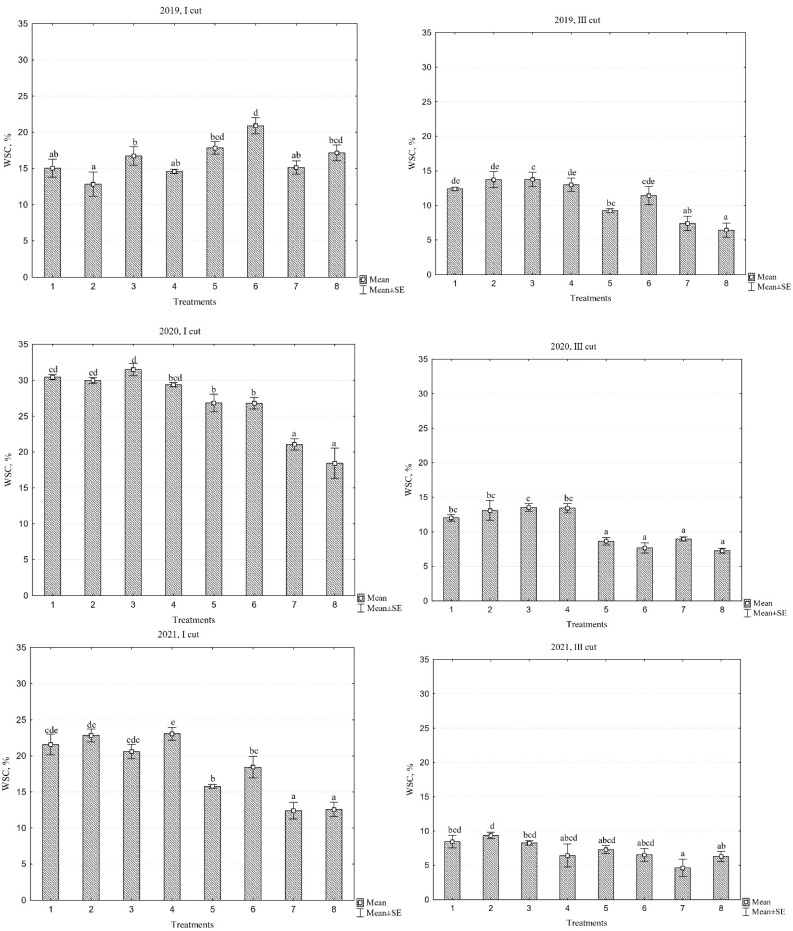
Water-soluble carbohydrates content across different cuts during the three-year period (2019–2021). Treatments: 1, perennial ryegrass (PR) cv. ‘Elena DS’; 2, PR cv. ‘Raminta’; 3, PR cv. ‘Verseka’; 4, PR cultivar mixture; 5, white clover + PR cv. ‘Elena DS’; 6, white clover + PR cultivar mixture; 7, white and red clover + PR cv. ‘Elena DS’; 8, white and red clover + PR cultivar mixture. Level of N fertilizer per year: 1–4 treatments (N_150_), 5–8 (N_0_); different letters indicate significant differences between the treatments (*p* < 0.05).

The WSC contents of the first and third cuts in 2019, 2020, and 2021 are shown in Figure 7. The data indicate that WSC levels in swards varied significantly depending on the harvest period. The first cut average annual WSC contents in 2019, 2020, and 2021 were 16.3%, 26.8%, and 18.4%, respectively, whereas in the third cut of each year, the WSC values were notably lower at 10.9%, 10.6%, and 7.2%. The second year of swards use, which provided the most favourable conditions for growth and development, exhibited the highest WSC compared to the other years. In the monoculture of perennial ryegrass (PR) cv. ‘Verseka’, the WSC content reached 31.5%, a value considerably above average, indicating superior forage quality. However, it is crucial to consider the overall nutritional profile, as the crude protein (CP) content in this sward was only 8.9%.

When examining the nutritional quality of individual cultivars, variations in WSC content were observed, though most of these differences were not statistically significant. Notably, in the first two years of sward use, PR ‘Verseka’ demonstrated higher nutritive value compared to swards sown with the cultivars ‘Elena DS’, ‘Raminta’, or a cultivar mixture. Additionally, swards containing only white clover exhibited higher WSC levels than those with both white and red clover, likely due to a greater proportion of grasses in these swards, but with lower CP content.

### 2.8. Dry Matter Digestibility

The data presented in Table 5 indicate that both the different species compositions of the swards and the different years had a significant effect on dry matter digestibility (DMD) content in the first cut, whereas no significant differences between the years were observed in the third cut. The DMD content in the first cut tended to increase over the years, averaging from 56.7% in the first year of sward use to 72.5% in the second year, and remaining at 70.7% in the third year. In contrast, in the third cut, the DMD content in the swards fluctuated insignificantly between 62.8% and 64.5%. The average data over the three years of the first cut indicate that the swards with diverse species composition, including PR ‘Elena DS’ and the PR cultivar mixture containing only white clover (WC), were highly digestible, averaging 68.4%. However, in many cases, the annual yield of these swards was significantly lower compared to the PR ‘Elena DS’/WC+RC sward, which had a DMD of 64.9%.

**Figure 8:**
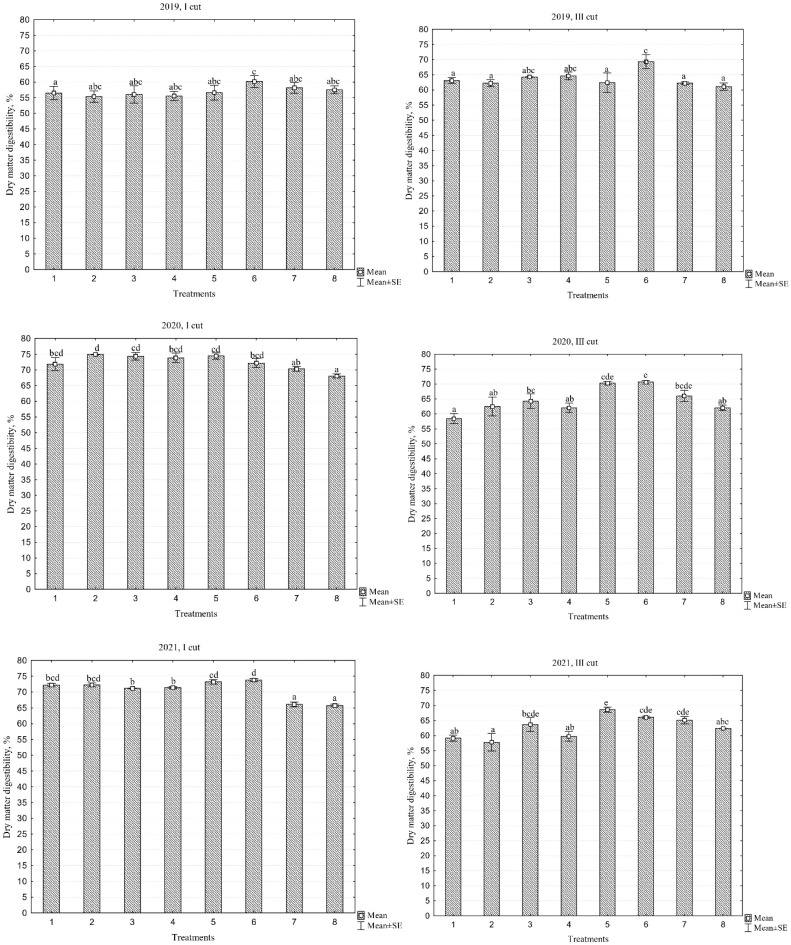
Dry matter digestibility across different cuts during the three-year period (2019–2021). Treatments: 1, perennial ryegrass (PR) cv. ‘Elena DS’; 2, PR cv. ‘Raminta’; 3, PR cv. ‘Verseka’; 4, PR cultivar mixture; 5, white clover + PR cv. ‘Elena DS’; 6, white clover + PR cultivar mixture; 7, white and red clover + PR cv. ‘Elena DS’; 8, white and red clover + PR cultivar mixture. Level of N fertilizer per year: 1–4 treatments (N_150_), 5–8 (N_0_); different letters indicate significant differences between the treatments (*p* < 0.05).

DMD indicates the proportion of dry matter in forage that can be efficiently digested and utilized by animals. A digestibility level above 70% is considered very good and indicates high forage quality, while levels below 60% typically suggest lower quality forage that is more difficult for animals to digest. Higher water-soluble carbohydrates (WSC) content is associated with higher DMD; WSC are easily digestible and rapidly available as an energy source, and their abundance directly contributes to the overall digestibility of forage. A high crude protein (CP) content is often associated with better forage quality and can help improve DMD, as proteins are essential for the digestion process. However, the correlation between CP and DMD is not always strong, as protein digestibility also depends on its source and the maturity of the forage; mature, fibrous forage may contain high protein levels, but be less digestible due to the high fibre content. Throughout the study period, the nutritional value of the swards remained high, except for the first cut in 2019 (Figure 8), where the DMD was slightly lower.

During the third cut in 2019, DMD values were higher, reaching nearly 70%, compared to the first cut, where the highest values were around 60%. This indicates that digestibility improved in the later cut. In 2020 and 2021, DMD values further increased, particularly during the first cut, where digestibility rose to 75% and 74%, respectively. This highlights a consistent trend of improving forage quality. Additionally, during the third cuts in both 2020 and 2021, the highest DMD values remained stable at around 70%, indicating that digestibility remained consistently high in later cuts.

## 3. Discussion

Perennial ryegrass (*Lolium perenne* L.) is the predominant forage grass cultivated in temperate regions [20], primarily due to its superior digestibility [21] and resilience to grazing pressures [22]. The key objectives in breeding programs for agricultural purposes focus on enhancing total and seasonal dry matter yield [23] across varying levels of fertilization, while improving digestibility, persistence [24], tolerance to freezing [25], and resistance to drought [26] and heat stress.

The hypothesis of this work was related to the fact that the integration of newer perennial ryegrass cultivars and cultivar mixtures, either fertilized with N_150_ or grown in combination with legume grasses, will result in higher forage productivity and improved nutritive value compared to monocultures. The combination of legumes enhances nitrogen use efficiency through symbiotic nitrogen fixation, thereby reducing the need for inorganic nitrogen inputs while sustaining or increasing forage yield and quality. Our study revealed differences in dry matter yield and quality parameters when evaluating individual PR cultivars; however, the cultivar mixture sward was not superior to the individual PR cultivars, a finding consistent with data reported by other researchers [17,19]. In particular, both perennial ryegrass and legumes such as white clover and red clover play crucial roles in contributing to overall forage quality in swards. Perennial ryegrasses offer several advantages when incorporated into species mixtures with legume grasses [27,28]. Our study showed that the newer PR cultivars with white clover (*Trifolium repens* L.) and red clover (*Trifolium pratense* L.) could exhibit improved yield and greater resistance to environmental stresses over years. Previous research has indicated that grass-legume mixtures combine high yields, low fertiliser requirements, and reduced nitrate leaching more effectively than either pure grass or pure legume swards, and this holds true both during the period with intact plant cover and after tillage for the subsequent crop [10].

However, when grown in combination with other legume grasses, newer grass cultivars can enhance the overall productivity and protein content of the mixture, as legumes fix atmospheric nitrogen, reducing the need for nitrogen fertilizers [29] and improving soil fertility [30]. Additionally, newer cultivars often demonstrate a better ability to compete with legumes without compromising digestibility or nutritional value, making the mixtures more efficient and sustainable. Furthermore, the inclusion of these improved perennial grass cultivars in mixtures enhances forage quality and nutritive value [31], leading to higher animal productivity [32], and can extend the grazing period by promoting consistent growth and yield across different seasonal stages.

Perennial ryegrass is valued for its high dry matter digestibility (DMD), rapid regrowth, and ability to maintain a relatively high forage yield across multiple seasons [33]. It also provides a readily available source of energy for grazing animals due to its high water-soluble carbohydrate (WSC) content, which enhances its palatability and improves overall animal performance [34]. Legumes, on the other hand, such as white and red clover [13], contribute significantly to the protein content of the forage due to their high crude protein (CP) levels. Additionally, legumes reduce the need for inorganic nitrogen fertilizers [16], which leads to more sustainable pasture systems [14]. They also improve soil fertility, enhance biodiversity, and can extend the grazing season by maintaining growth under conditions where grasses might slow down, such as during dry periods. The combination of grasses and legumes in mixed swards improves the balance between energy from grasses and protein from legumes, leading to higher forage quality and better animal performance.

It is also important to note that the effective symbiosis between legumes and rhizobia, as well as yield, forage quality, biological nitrogen fixation, and other significant parameters, are determined by the physiological and genetic characteristics of both symbionts, along with soil and meteorological conditions. However, the most critical factors are soil acidity and the concentration of mineral nitrogen compounds in the soil. High rates of mineral nitrogen fertilizers alter protein composition, increase non-proteinogenic amino acids, modify plant hormonal status, reduce membrane permeability, and slow the movement of assimilates within the plant [35]. As a result, this study, in line with others [11,36], demonstrated that legumes in mixtures can replace the use of mineral nitrogen, thereby fulfilling ecosystem service functions.

As expected, the productivity of swards varied over the years due to their age, which is consistent with the results reported by other researchers [37]. However, in our experiment, the decline in productivity with increasing sward age did not follow a consistent trend due to the drought conditions that occurred during the first year of sward use, reaching the thresholds of hydrological drought. These conditions resulted in a 19.7% average reduction in overall swards biomass compared to the second year of use. During the first year, drought stress prevented grasses from establishing properly due to the lack of water necessary for root system development, which can impede their ability to absorb water and nutrients in subsequent years and affect long-term productivity [38]. Moreover, in such a situation, grasses may deplete their energy reserves, which are essential for regeneration and growth in the following seasons. This can reduce their vitality and resilience to future environmental stress [39]. Different species and cultivars respond differently to drought conditions, and newly developed drought-tolerant cultivars may be more suitable for mixed swards to ensure long-term yield stability [37].

Our study data showed that in the second year of sward use, the average productivity increased to 8052 kg ha^−1^, with a specific sward containing PR ‘Elena DS’ showing a 15.9% increase to 8699 kg ha^−1^, and PR ‘Elena DS’/WC+RC mixtures achieving an even greater 46% increase to 10,308 kg ha^−1^ compared to the first year. This demonstrates the ability of drought-tolerant cultivars to recover more effectively after drought conditions, similar to the findings of [39] In Lithuania’s climate, the average productivity of cultivated grasslands can range from 5 to 8 t of DM ha^−1^ per season, depending on the technologies and fertilization levels applied. In highly productive grasslands, with proper fertilization and advanced management practices, yields can reach as much as 10 to 12 t of DM ha^−1^ per season. Our research results reflect the potential of achieving high productivity in swards where compatible species and cultivars are selected and grown together in mixtures.

## 4. Materials and Methods

### 4.1. Experimental Site

A field experiment was carried out from 2018 to 2022 at the Lithuanian Research Centre for Agriculture and Forestry, located in Akademija, Central Lithuania (55°22′59.7″ N, 23°51′42.1″ E). The soil at the site, classified as endocalcaric epigleyic cambisol [40], is derived from loamy sand. Before the experiment began in 2018, the arable soil layer (0–25 cm) showed a high content of available macronutrients, with phosphorus (P) at 98 mg kg^−1^ and potassium (K) at 144 mg kg^−1^. Organic carbon (C_org_) levels were relatively high at 1.76%, and total nitrogen (N) was measured at 0.247%. The soil pH was neutral, with a value of 6.9.

### 4.2. Weather Conditions

**Figure 9:**
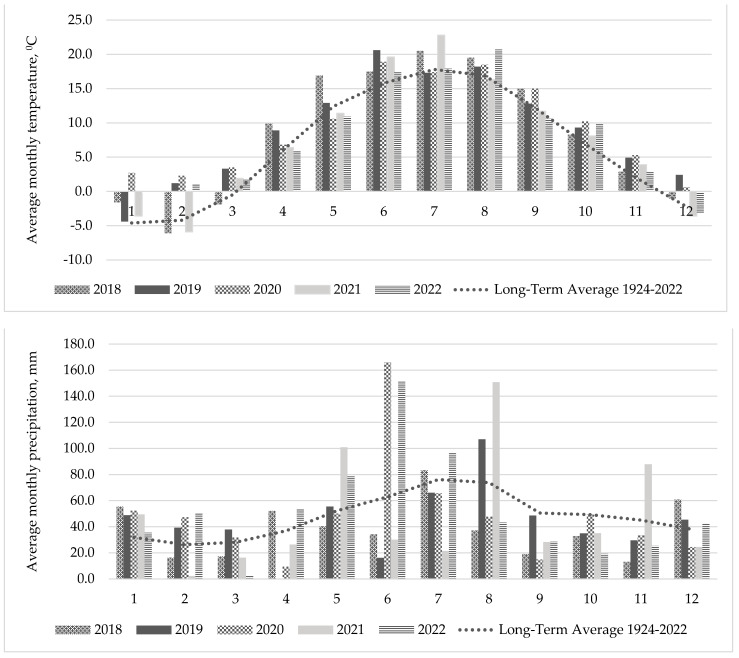
Average monthly air temperature and precipitation from 2018 to 2022.

The climatic conditions fluctuated significantly throughout the duration of the experiment. In this context, Figure 9 presents the average monthly air temperature and total precipitation over the study period. The meteorological data employed in this study were sourced from the Dotnuva meteorological station, situated less than three kilometres from the experimental site. Notably, according to the Lithuanian Hydrometeorological Service, the average annual air temperature in Lithuania has remained at 7.4 °C since 2021, based on the climate norms established from 1991 to 2020. Furthermore, this period has witnessed an average annual precipitation of 675 mm. In comparison to the previous climate norm (1981–2010), there has been a 3% decrease in average annual precipitation, along with a 0.4 °C increase in average air temperature.

The new indicators demonstrate a clear increase in average temperatures. Notably, a significant rise in temperature has been observed during the warm season, particularly in summer, as well as during the milder winter months. Thus, these changes indicate a significant shift in climatic conditions that could impact agricultural practices and ecosystem dynamics in Lithuania.

At the outset of the experiment, the year of sowing exhibited favourable conditions for plant growth, characterized by high humidity and elevated temperatures, which facilitated optimal germination and swelling. Conversely, the years 2019 and 2021 were particularly unfavourable for plant growth, primarily due to reduced precipitation and above-average temperatures. During the summer of 2022, the weather was predominantly windy, warm, and rainy, except for August. The average air temperature for the summer months was 1.8 °C above the long-term average of 16.8 °C from 1924 to 2022. Total precipitation amounted to 297.5 mm, representing 140% of the 1924–2022 average (212.1 mm). A significant rainfall period was recorded in the first and second decades of June, while August was notably dry. Overall, the weather conditions were relatively favourable for the growth and development of swards.

### 4.3. Experimental Design

The temporary grassland field experiment was sown in 2018, and during this year, only cleaning cuts were performed in the swards for weed control, while 2019, 2020, 2021, and 2022 represented the first, second, third, and fourth years of sward use, respectively. In the spring of 2018, before sowing, NPK fertilizers were applied at a rate of N–P–K 5–20.5–36 kg ha^−1^. During all subsequent years, single-species swards received mineral nitrogen fertilizers at a rate of 150 kg N ha^−1^ per year: N60 in spring, followed by N45 after both the first and second cuts. The swards were harvested four, five, four, and three times in 2019, 2020, 2021, and 2022, respectively. Grass-legume mixtures did not receive any fertilization. The site was managed without additional irrigation, and no pesticides were applied. To determine the productivity of the sward, the plants were cut according to the dominant plant species in the sward at the beginning of the heading stage of the grasses. The sowing rates for single-species swards and mixtures, composed of Lithuanian cultivars, namely, white clover (*Trifolium repens* L.) cv. ‘Dotnuviai’, red clover (*Trifolium pratense* L.) cv. ‘Sadūnai’, and perennial ryegrasses (*Lolium perenne* L.) cv. ‘Elena DS’, ‘Raminta‘, and ‘Verseka’, were 10, 15, and 18 kg ha^−1^, respectively. In the mixtures, the seed rate was adjusted according to the species composition, with 40% legumes and 60% grasses (Table 6). The swards were sown in plots of 15 m^2^ (1.5 × 10 m) each.

Mineral nitrogen fertilization (150 kg N ha^−1^ per year) was used from the first year of sward use.

### 4.4. Plant Sampling and Measurements

The number of harvests varied across the years, depending on the growing conditions, with four, five, four, and three cuts made in 2019, 2020, 2021, and 2022, respectively. To assess sward biomass and botanical composition and quality (except in 2022), individual samples (1–1.5 kg) were collected. The fresh biomass was immediately weighed to determine the fresh weight. For dry matter yield (DMY) determination, the fresh biomass was oven-dried at 105 °C until a constant weight was reached, and then weighed again. To assess the botanical composition, the plants in each sample were separated by species, including grasses, legumes, and forbs. Each plant species was weighed separately, and the obtained weight data were used to calculate the percentage contribution of each species to the total biomass. To prevent alterations in the chemical composition, these samples were immediately dried at 60 °C for 48 h in a well-ventilated oven. Once dried, the samples were ground and prepared for chemical analysis. The potential of near-infrared reflectance spectroscopy (NIRS) for determining the chemical composition of sward herbage was evaluated. Samples were collected over four consecutive years from different cuts and analyzed using the NIRS method for crude protein, modified acid detergent fibre (MADF), neutral detergent fibre (NDF), water-soluble carbohydrates (WSC), and dry matter digestibility (DMD).

### 4.5. Statistical Analysis

Data were analyzed using a two-way ANOVA with two factors: (1) different combinations of plant species, and (2) different years, including their possible interactions. A one-way ANOVA was applied to compare treatments within each year. The least significant difference (LSD) test was used to estimate differences between treatments for all parameters at the 5% significance level (*p* < 0.05). All analyses were performed using STATISTICA 8, and the results are presented as mean values with their standard errors (SE).

## Figures and Tables

**Table 1:** Significance of crude protein in aboveground biomass of different combinations of plant species swards in 2019–2021.

Year	2019		2020		2021	
Cuts	I	III	I	III	I	III
Factor A (grass–legume mixtures)	*	*	*	*	*	*
Factor B (years)	*	*	*	*	*	*
Interaction A × B	*	*	*	*	*	*

* Values with asterisks indicate significant differences (* *p* < 0.05).

**Table 2:** Significance of modified acid detergent fibre in aboveground biomass of different combinations of plant species swards in 2019–2021.

Year	2019		2020		2021	
Cuts	I	III	I	III	I	III
Factor A (grass–legume mixtures)	*	*	*	*	*	*
Factor B (years)	*	*	*	*	*	*
Interaction A × B	*	*	*	*	*	*

* Values with asterisks indicate significant differences (* *p* < 0.05).

**Table 3:** Significance of neutral acid detergent fibre in the aboveground biomass of different combinations of plant species swards in 2019–2021.

Year	2019		2020		2021	
Cuts	I	III	I	III	I	III
Factor A (grass–legume mixtures)	*	*	*	*	*	*
Factor B (years)	*	ns	*	ns	*	ns
Interaction A × B	ns	*	ns	*	ns	*

* Values with asterisks indicate significant differences (* *p* < 0.05), and ns indicates non-significant difference.

**Table 4:** Significance of water-soluble carbohydrates in aboveground biomass of different combinations of plant species swards in 2019–2021.

Year	2019		2020		2021	
Cuts	I	III	I	III	I	III
Factor A (grass–legume mixtures)	*	*	*	*	*	*
Factor B (years)	*	*	*	*	*	*
Interaction A × B	*	*	*	*	*	*

* Values with asterisks indicate significant differences (* *p* < 0.05).

**Table 5:** Significance of dry matter digestibility in aboveground biomass of different combinations of plant species swards in 2019–2021.

Year	2019		2020		2021	
Cuts	I	III	I	III	I	III
Factor A (grass–legume mixtures)	*	*	*	*	*	*
Factor B (years)	*	ns	*	ns	*	ns
Interaction A × B	*	*	*	*	*	*

* Values with asterisks indicate significant differences (* *p* < 0.05), and ns indicates non-significant difference.

**Table 6:** The composition of single-species swards and mixtures with legumes; the numbers in the columns indicate the targeted proportion of the species at sowing.

Treatments	FG	SP-r	FG of Grasses	FG of Legumes	Perennial Ryegrass			Clover		
					Elena DS G1	Raminta G2	Verseka G3	Dotnuviai L1	Sadūnai L2	N-Level
1	1	1	1		1					N_150_
2	1	1	1			1				N_150_
3	1	1	1				1			N_150_
4	1	1	1		0.33	0.33	0.33			N_150_
5	2	2	0.6	0.4	0.6			0.4		N_0_
6	2	2	0.6	0.4	0.2	0.2	0.2	0.4		N_0_
7	2	3	0.6	0.4	0.6			0.2	0.2	N_0_
8	2	3	0.6	0.4	0.2	0.2	0.2	0.2	0.2	N_0_

## Data Availability

Data are contained within the article.
